# Effects of Dysphagia Therapy on Swallowing Dysfunction after Total Thyroidectomy

**DOI:** 10.22038/ijorl.2019.36233.2193

**Published:** 2019-11

**Authors:** Mohadeseh Hashemian, Bijan Khorasani, Maryam Tarameshlu, Hamid Haghani, Leila Ghelichi, Noureddin Nakhostin Ansari

**Affiliations:** 1 *Department of Speech and Language Pathology, School of Rehabilitation Sciences, Iran University of Medical Sciences, Tehran, Iran.*; 2 *Department of Clinical Sciences, University of Social Welfare and Rehabilitation Sciences, Tehran, Iran. *; 3 *Rehabilitation Research Center, Department of Speech and Language Pathology, School of Rehabilitation Sciences, Iran University of Medical Sciences, Tehran, Iran.*; 4 *Department of Biostatics, School of Management and Information, Iran University of Medical Sciences, Tehran, Iran.*; 5 *Rehabilitation Research Center, Department of Speech and Language Pathology, School of Rehabilitation Sciences, Iran University of Medical Sciences, Tehran, Iran.*; 6 *Department of Physiotherapy, School of Rehabilitation, Tehran University of Medical Sciences* *, Tehran, Iran* *.*

**Keywords:** Deglutition, Traditional dysphagia therapy, Thyroidectomy, Swallowing disorders

## Abstract

**Introduction:**

Swallowing disorder or dysphagia is a common complication after conventional total thyroidectomy. Traditional dysphagia therapy (TDT) has long been a routine rehabilitation program for patients with dysphagia; however, there is no evidence to support the efficacy of this approach in patients with post-thyroidectomy dysphagia. Regarding this, the purpose of the current study was to explore the effectiveness of TDT in swallowing dysfunction in patients suffering from post-thyroidectomy dysphagia.

**Materials and Methods:**

This pilot clinical trial was conducted on 21 patients with post-thyroidectomy dysphagia. The study population was randomly assigned into two groups of TDT and control. The patients in the TDT group received 18 treatment sessions for 6 weeks, 3 times a week. The Swallowing Impairment Score (SIS-6), Functional Oral Intake Scale (FOIS), and Persian Dysphagia Handicap Index (P-DHI) were the outcome measures. The outcome variables were assessed at the baseline, at the end of the treatment, and after a 6-week follow-up. The main effects of time and group and their interaction effect on SIS-6 and P-DHI scores were examined using repeated measures ANOVA. In addition, the intergroup comparison in terms of the FOIS score was analyzed using the Mann-Whitney U test. The Cohen's d effect size was also measured to ascertain the effects of the treatment.

**Results:**

According to the results, the TDT group showed a significant improvement in the SIS-6, FOIS and P-DHI scores over time (P<0.001). The results also revealed that the interaction effect of time and group was significant on SIS-6 and P-DHI scores (P<0.001). In addition, effect sizes on SIS-6, FOIS, and P-DHI scores were large in the TDT group.

**Conclusion:**

This study suggested that TDT could improve the swallowing dysfunction in the patients suffering from post-thyroidectomy dysphagia. As the results indicated, the improvements persisted 6 weeks after the end of TDT.

## Introduction

Thyroidectomy is the most frequent endocrine operation in the world (1). The common clinical outcomes following thyroidectomy include dysphonia and dysphagia, hemorrhage, hypocalcemia, hypoparathyroidism, damaged recurrent laryngeal nerve, and damaged superior laryngeal nerve (1-6). Dysphagia or swallowing dysfunction is commonly seen in patients with thyroidectomy and has a prevalence range of 20-58% (1,2,^7^-^9^).

Several factors can cause post-thyroidectomy dysphagia, including cricopharyngeal dysfunctions (10), intraoperative manipulation, retraction of the scar tissue, thyroid gland palpation (6,11), injury of the neural plexus, tracheal intubation, reaction to pain, changes in the blood supply (2,4), strap muscle malfunction, laryngotracheal fixation, psychological reactions to surgery, and adhesion between the subplatysmal muscle flap and the strap muscle (4,12).

Some symptoms of the post-thyroidectomy dysphagia include non-specific swallowing changes, painful or difficult swallowing, sense of a lump, and coughing (4,13,14). Dysphagia increases the risk of malnutrition, dehydration, and aspiration pneumonia; moreover, this condition can lead to the reduction of patients’ quality of life and sudden death in patients with thyroidectomy (15-17). Therefore, post-thyroidectomy dysphagia as a serious condition should be diagnosed and managed with proper therapeutic strategies that are based on comprehensive clinical evaluations. 

The main objective of dysphagia therapy is to achieve normal nutrition (18). Traditional dysphagia therapy (TDT) as a common rehabilitation technique includes the modification of the diet, compensatory techniques, motor exercises, and swallowing maneuvers (18). There is evidence regarding the efficacy of TDT in the swallowing performance of the dysphagic patients due to different reasons, such as multiple sclerosis, Parkinson's disease, head and neck cancer, and stroke (19-23). However, there is a dearth of published studies relevant to the effectiveness of TDT on the swallowing dysfunction of the patients with thyroidectomy. Regarding this, the purpose of the current pilot research was to evaluate the effectiveness of TDT on swallowing dysfunction in patients suffering from post-thyroidectomy dysphagia.

## Materials and Methods

This pilot study was targeted toward the investigation of the effectiveness of TDT in swallowing dysfunction after conventional total thyroidectomy (CTT). The protocol of this research was approved by the Ethical Committee of Iran University of Medical Sciences, Tehran, Iran.

**Study population**

This study was conducted on patients undergoing CTT at the Surgical Ward of Milad Hospital, Tehran, Iran, between November 2016 and May 2017. The inclusion criteria included: 1) age range of 21-60 years, 2) infliction with dysphagia based on the swallowing impairment score (SIS-6) (24), 3) implementation of CTT at least one month prior to the study, 4) lack of alcohol consumption or smoking, 5) lack of vocal fold paralysis, and 6) lack of a previous neck surgery. On the other hand, the exclusion criteria were: 1) pulmonary or neurological diseases, 2) history of severe reflux, and 3) dysphagia due to drug toxicity. The patients were asked to sign an informed consent before initiating the study.

**Intervention**

The patients were randomly allocated into two groups of TDT (n=11) and control (n=10). The TDT group received TDT, and the control group did not receive any dysphagia therapy. All the patients in the TDT group were subjected to 18 treatment sessions, 3 times a week. The patients were demanded not to take part in any other swallowing intervention programs. The TDT program included diet modification, compensation strategies, oral motor exercises (i.e., laryngeal elevation, masako or tongue hold, and shaker exercise), postural correction to facilitate bolus transition (e.g., chin-down posture, chin-up posture, head rotation, and head tilt), thermal tactile stimulation, and mendelsohn, supraglottic, super supraglottic and effortful maneuvers (18).

**Outcome measures**

At first, the patients underwent a baseline interview to collect the demographic data by a speech-language pathologist. Then, the SIS-6, Functional Oral Intake Scale (FOIS), and the Persian version of the Dysphagia Handicap Index (P-DHI) scores were documented at the baseline, at the end of the treatment, and after a 6-week follow-up period. In this study, the SIS-6, FOIS, and P-DHI were used as the outcome measures. These scales are validated clinical questionnaires facilitating the diagnosis of swallowing dysfunctions (24-27). 

**Swallowing impairment score**

The SIS-6 is a self-evaluation questionnaire assessing common dysphagia symptoms. The SIS-6 includes 6 items scored within a range of 0 (without swallowing alterations) to 24 (maximum swallowing dysfunction). The SIS-6 is a validated tool for dysphagia diagnosis (14,24).

**Functional Oral Intake Scale**

The FOIS is a seven-point scale to determine the functional degree of oral feeding in stroke patients. Levels 1-3 relate to different levels of non-oral feeding, and levels 4-7 denote different levels of oral intake. The FOIS scale has demonstrated good reliability and validity in assessing functional oral feeding (25).

**Dysphagia Handicap Index**

The P-DHI consisting of 25 items was used to measure the handicapping impact of swallowing disorders. The reliability and validity of the questionnaire have been already demonstrated in previous studies (26). In this instrument, the patient should select one of the three options of ‘always’, ‘sometimes’, and ‘never’ for each item scored 0, 2, and 4, respectively. The total score is obtained by summing up all item scores and ranges from 0 to 100 (26,27).

*Statistical Analysis*

The statistical analysis of the data was performed in SPSS software (version 19; Inc., Chicago, IL, USA). Normality of the data was determined using the Kolmogorov-Smirnov test. The repeated measures ANOVA was applied to evaluate the main effects and interaction effects of time and group on the SIS-6 and P-DHI scores. In addition, the Bonferroni test was performed for posthoc pairwise analysis. The intergroup comparison of FOIS scores was determined by means of the Mann-Whitney U test. Furthermore, the Friedman test was used to perform the intragroup comparison of the FOIS score. The Cohen's d effect sizes were also measured to ascertain the effects of treatment. A p-value of ≤ 0.05 was considered statistically significant.

## Results

**Fig 1:**
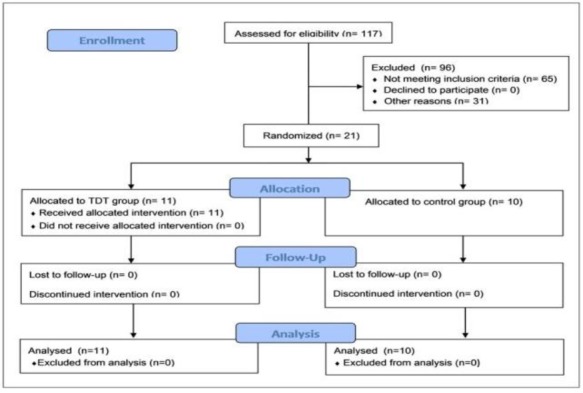
Consort flow diagram

Figure 1 displays a consort flow chart of the participants. Out of the 117 patients who underwent CTT, 21 patients (i.e., 4 males and 17 females) with a mean age of 46±9 years met the inclusion criteria. The study population was randomly assigned into two groups of TDT (n=11) and control (n=10). All participants completed the study procedure. According to the results, 5(23%), 12(57%), and 4(19%) patients had thyrotoxicosis, thyroid carcinoma, and goiter, respectively (Table.1). The characteristics and outcome measures did not significantly differ between the two groups at the baseline (Table.1).

**Table 1:** Characteristics of the patients at baseline in Traditional Dysphagia Therapy (TDT) and Control groups

	**TDT Group (N = 11)**	**Control Group (N = 10)**	**Test (P–value)**
Female/male	8/3	9/1	Chi-square test (0.58)
Mean age ± SD (years)	49.27±7.36	42.40±10.22	Independent T-test 0.09
Preoperative diagnosis			Chi-square test (0.4)
Thyrotoxicosis	3(14%)	2(9%)	
Thyroid carcinoma	6(28.5%)	6(28.5%)	
Goiter	2(9.5%)	2(9.5%)	
SIS-6 (Mean ± SD)	14.27 ± 2.1	13.9 ± 2.7	Independent T-test (0.73)
FOIS (Median , Interquartile Range)	5 (4-6)	5 (5-6)	Mann-Whitney U test (0.34)
P-DHI (Mean ± SD)	42.55 ± 8.6	39.20 ± 6.4	Independent T-test (0.33)

TDT: traditional dysphagia therapy, SIS-6: swallowing impairment score, FOIS: Functional Oral Intake, P-DHI: Persian version of the dysphagia handicap index score

**Swallowing Impairment Score**

**Fig 2:**
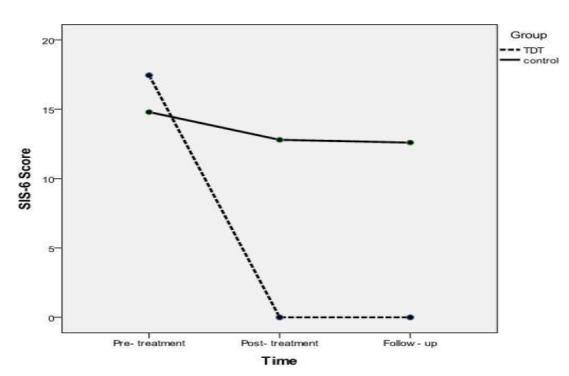
Interaction effect of time and group on swallowing impairment score

According to the results of the Mauchly’s test, time showed a significant effect on SIS-6 (χ2=38.11, df= 2, P<0.001; F(1.06, 20.22)=145.77, P<0.001, Greenhouse-Geisser adjusted). The results of the Bonferroni posthoc test showed a significant improvement in the SIS-6 score in the TDT group at the end of the treatment (P<0.001) and after the follow-up period (P<0.001). The SIS-6 score improvement retained 6 weeks after the treatment (P=0.19). The main effect of groupon SIS-6 was also found to be significant (F_(1,19) _=25.79, P<0.001). In addition, the time and group interaction effect on SIS-6 score was significant; (F_(__1.06,20.22__.)_=77.81, P<0.001, Greenhouse-Geisser adjusted; Fig.2). Effect sizes on the SIS-6 were also large (d=7.64) and small (d=0.46) in the TDT and control groups, respectively.

**Functional Oral Intake Scale**

As the results indicated, the FOIS score was significantly improved over time in the TDT group ($\chi 2\left(2\right)=$26, P<0.001). There was a significant difference between the two groups in terms of the FOIS score after the treatment and 6 weeks after the follow-up($\chi 2\left(1\right)$=13.20, P<0.001). In this regard, the TDT group showed an improvement in this variable. Effect sizes on the FOIS score for the TDT and control groups were large (d=1.41) and small (d=0.00), respectively.

**Persian version of the Dysphagia Handicap Index**

**Fig 3:**
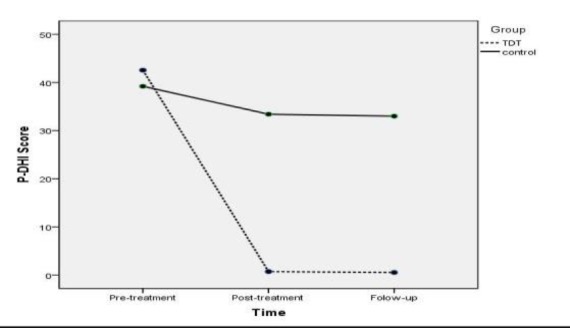
Interaction effect of time and group on the Persian version of the dysphagia handicap index score

Based on the results of the Mauchley’s test, time significantly affected the P-DHI score (χ2=71.64, df=2, P<0.001; F_(1.01,20.28)_=105.65, P<0.001, Greenhouse-Geisser adjusted). Furthermore, the results of the Bonferroni posthoc test revealed a significant improvement in the P-DHI score of the TDT group after the end of the treatment (P<0.001) and follow-up period (P<0.001). The P-DHI improvement retained 6 weeks after the treatment (P=0.34). The significant main effect of group was also observed on the P-DHI (F_(1,19) _=29.49, P<0.001). In addition, the time and group interaction effect on P-DHI score was found to be significant (F_(1.01,20.28.)_=59.35, P<0.001, Greenhouse-Geisser adjusted; Fig.3). Effect sizes on the P-DHI score were large (d=6.83) and small (d=0.49) for the TDT and control groups, respectively.

## Discussion

The findings of the current study revealed an improvement in the SIS-6, P-DHI, and FOIS scores of the TDT group over time. Furthermore, the improvement of SIS-6, P-DHI, and FOIS scores persisted during the 6 weeks of follow-up. To the best of our knowledge, the present research is the first attempt toward the investigation of the effectiveness of TDT in swallowing dysfunction among the patients suffering from post-thyroidectomy dysphagia.

**Swallowing Impairment Score**

The results of our study indicated that the SIS-6 score improved after 18 sessions of treatment and that this improvement lasted 6 weeks after the treatment in the TDT group. Moreover, time and group were demonstrated to exert an interactive effect on the SIS-6 score; in this regard, the improvements in the TDT group was more than that in the control group over the course of time. The lack of similar studies hinders the comparison of our findings. The results indicated that TDT can be efficient in modifying the weakness of the muscles, muscle tonicity, coordination, and reduced endurance (7,18,22). In addition, a large effect size was observed on the SIS-6 score of the TDT group after the treatment. This finding suggests that the TDT was greatly efficient in the improvement of swallowing dysfunction in patients suffering from post-thyroidectomy dysphagia.

**Functional Oral Intake Scale**

The findings of this study revealed that the FOIS score was improved after 18 therapeutic in the TDT group. Furthermore, the FOIS improvements persisted 6 weeks after the treatment. All patients in the TDT group regained normal feeding without any restrictions. These results suggest the higher safety of oral feeding after treatment in the TDT group (18,23,24). A large effect size on FOIS score was seen at the end of the 18 sessions of the treatment in the TDT group. These findings suggest the efficiency of TDT in the improvement of oral feeding in patients with post-thyroidectomy dysphagia.

**Persian version of the Dysphagia Handicap Index**

The results of the current study were indicative of an improvement in the P-DHI score of the TDT group after 18 sessions of treatment, which lasted six weeks after treatment. Moreover, the interactive effect of time and group was shown on the P-DHI; in this respect, the improvements of the P-DHI score in the TDT group was more than that in the control group over time. These findings imply that TDT exercises increase the quality of life via improving swallowing abilities (18,24). Our study showed large effect sizes on P-DHI score at the end of 18 sessions of treatment in the TDT group. These findings indicate that TDT was greatly effective in reducing the handicapping impacts of swallowing problems in patients suffering from post-thyroidectomy dysphagia.

This study has some limitations that should be noted. First, the sample size was small which could limit the generalizability of the results. Second, there was no blinding for patients, dysphagia therapist, or assessor. Therefore, it is recommended to perform well-designed randomized controlled trials with large sample size to confirm our findings.

## Conclusion

The findings of the present pilot study revealed the efficiency of TDT in the improvement of swallowing dysfunction in patients suffering from thyroidectomy. However, further studies are required to compare the efficacy of TDT with those of other treatments in these patients.
